# Effect of drill cuttings addition on physicochemical and chemical properties of soil and red clover (*Trifolium pretense* L.) growth

**DOI:** 10.1371/journal.pone.0242081

**Published:** 2020-11-12

**Authors:** Justyna Kujawska, Małgorzata Pawłowska

**Affiliations:** Faculty of Environmental Engineering, Lublin University of Technology, Lublin, Poland; Universidade de Santiago de Compostela, SPAIN

## Abstract

The most economical method of drill cuttings disposal may be their application in land reclamation which allows for the wastes recovery. However, the wastes application into the soil should ensure that the quality of the environment would not be deteriorated. These investigations were aimed at identifying the effect of drill cuttings, which were the mixture of different types of drilling wastes, on the physicochemical properties of acidic soil and growth of red clover (*Trifolium pratense* L.). The experimental design comprised 5 treatments, which differed in a dose of the drill cuttings: 0% (control), 2.5%, 5%, 10% and 15% of dry weight. A six-week pot experiment was conducted to determine the influence of the wastes on the plant growth. The results showed that the drill cuttings addition significantly changed the chemical and physicochemical properties of the soil, such as: electrical conductivity (EC), pH, base saturation, content of carbonate, alkaline cations (Ca^2+^, Na^+^, K^+^, Mg^2+^), organic matter, total organic carbon (TOC), and available phosphorus form. However, the most important factors that influenced the growth of red clover were pH, base saturation, content of Mg^2+^ and plant available phosphorus. The red clover biomass was increased from 1.5 to 2.5 times depending on the dose of wastes. We concluded that the examined wastes can be used for reclamation of the acid and unfertile degraded soils, but the amount of wastes should not exceed 5% of the soil, because the highest total clover biomass was observed just at this dose.

## Introduction

Drilling wastes are generated during extraction of fossil fuels from the lithosphere. The majority of drilling wastes consists of geological material. In comparison with the wastes produced in other branches of industry, the drilling wastes are usually considered as the ones with low volume and toxicity. However, since the conventional resources of crude oil and natural gas are becoming depleted, the new methods, including horizontal drilling, and increase in the depth of drilling cause gradual growth in the volume of wastes. For example, in Poland, the production of drilling wastes in 2010 amounted to 47.6 thousand tons [1] including 10.5 thousand tons of waste derived from the shale gas exploration processes, while over 50.8 thousand tons of the wastes were produced in 2012 [2]. The Polish Geological Institute estimated that between 2013 and 2017, the annual generation of drilling wastes in Poland reached 87.5 thousand tons [3].

In recent years, the legal regulations pertaining to environmental protection around the world have become increasingly stringent. The conventional approach to wastes processing is insufficient; hence, the methods involving the reuse of drilling wastes are being introduced. The search for more economical, efficient and safer methods of drilling wastes processing is still a relevant topic.

Drilling wastes may be reused as a secondary component, especially in civil engineering as building filler [4–6]. Other methods of drilling wastes management include thermal techniques (incineration, thermal desorption), biological methods (e.g. composting, co-treatment with wastewater) [7–9], as well as surface or underground depositing (e.g. reinjection) [10, 11]. However, the proposed solutions require further studies taking into account the environmental requirements, as well as the economic and energetic aspects [12].

Introduction of drilling wastes to soils, as well as landfarming and landspreading processes are widespread in the United States of America. The legal regulations in the European Union do not allow the application of drilling wastes onto the land surface, in contrast to the law in Canada or the United States. Thus, in Poland, these methods are not employed [13], although adding drilling wastes to soils seems to be a convenient method of their management.

The drill cuttings are characterized by high cation exchange capacity (CEC). Their introduction to soils improves the capacity to retain cations, which was confirmed in the studies conducted on sandy soils [14]. An increase in the value of pH and EC in the soils following the drilling wastes addition was noted by numerous scholars [14–18]. However, McFarland et al. [1994]; Miller et al. [1980]; Zvomuya et al. [2009, 2008] observed a negative impact of spent water-based drilling muds on the soil processes and plant growth, attributing these effects to the high concentration of soluble salts or heavy metals [19–22]. Identifying the potential influence of wastes addition on the plant growth is important not only in terms of determining the susceptibility of plants to drill cuttings, but also determining the pollutants contained in their biomass [23]. Various levels of susceptibility of different plants (e.g. grasses, cereals, legume) to the presence of pollutants in the soil with drilling wastes are presented in literature [14–18, 22, 24–29].

The aim of the study was to determine the maximum dose of drilling wastes that can be added to acidic degraded soils without causing the negative changes in the physicochemical properties of the soil and plant growth. The practical aim was to evaluate the feasibility of managing these waste for a land reclamation purpose.

We assumed that an addition of the wastes to acidic soil, in which silt is a predominant fraction in their granulometric composition, the mixture of drilling wastes with different composition and origin may improve the physical, chemical and physicochemical properties of the soil and increase in the yield of plant biomass, but this positive effect will be reduced when the threshold dose is exceeded. In order to achieve the aim of the study, four types of soil-waste mixtures were subjected to physicochemical analysis and the impact of the drill cuttings addition on the plant growth was determined through a pot experiment.

## Methods and materials

The marginal mineral soil was taken from the arable field in the vicinity of Lublin, fallow for many years, because of infertility, from a depth of 10–20 cm. The owner of the land gave us permission to take soil samples for the laboratory study. No specific permissions were required for these locations and the studies did not involve endangered or protected species.

The soil was acidic (pH 4.2 in 1 M KCl) and poor in organic substances (the content of organic matter was 0.7% of dry weight), nitrogen (0.06% of dry weight) and macronutrients available for plants (P– 5.79 mg P_2_O_5_ 100 g^-1^ and K– 2.18 mg K_2_O 100 g^-1^). It was classified as silt loam according to USDA standards [30]. The drilling wastes were obtained at the site of their final management, in a facility located in the south-eastern Poland. The wastes used in the study were a mixture of different types of residues produced during the exploration of oil and natural gas in southern Poland, but their detail origin and composition are unknown. Before deposition, the wastes were mixed with coagulant–aluminium sulphate (up to few percent of dry weight), dewatered in chamber filter press, mixed with cement (up to few percent of weight) and in such form used for the technical reclamation of open mining pit. The characteristics of drill cuttings were given in Table 1.

**Table 1:** Physicochemical properties of drill cuttings.

Parameters	Drill cuttings
Granulometric composition	
• sand fraction [%]	75±9
• silt fraction [% of weight]	18±8
• clay fraction [% of weight]	7±3
Specific gravity [g/cm^3^]	2.62±0.02
Bulk density [g/cm^3^]	1.02±0.09
Porosity [%]	60.68±2.76
Carbonate content [%]	36.22±3.60
Total exchangeable bases–TEB [cmol(+)/kg]	49.50±0.24
Hydrolytic acidity–H [cmol(+)/kg]	0.15±0.01
Cation exchange capacity–CEC [cmol(+)/kg]	49.65±1.74
Organic matter [% of dry weight]	11.52±0.21
pH	9.6±2.31
Electrical conductivity (EC) [mS/m]	40.1±2.68
N tot. [% of dry weight]	0.04±0.001
TOC [% of dry weight]	6.8±0.33

### Pot experiment

The studies were carried out on four types of soil-waste mixtures, in which the share of cuttings calculated by weight (based on air dried weight) was as follows: 2.5% (Z-2.5), 5% (Z-5), 10% (Z-10), and 15% (Z-15). The control sample (Z0) test was the soil without drill cuttings (Table 2). Each soil substrate was examined in three replications. The soil and waste samples were air dried and sieved (2 mm) before there were used for mixtures preparing.

**Table 2:** Composition of the soil substrates used in the pot experiment.

Soil substrate	Share in the substrate	
	(% of “air dried” mass)	
	Drill cuttings	Soil
**Z0 (control)**	0	100
**Z-2.5**	2.5	97.5
**Z-5**	5	95
**Z-10**	10	90
**Z-15**	15	85

The materials were placed in plastic pots with a capacity of 350 mL and each of them was sown with 12 red clover seeds (*Trifolium pratense* L.). The studies were carried out in 3 replicates, for 6 weeks. The experiment was carried out in a phytotron with a 13/11 light/dark regime. At the daytime (7 am—8 pm) air temperature was 25°C while the night-time (8 pm—7 am) air temperature was 16°C. During the experiment, the plants were watered with distilled water. The amount of water depended on the current needs of the plants. After the experiment, the plants were harvested and the roots were gently separated from the soil by repeated rinsing. The collected biomass of the plants were divided into roots and above-ground (shoots) parts. Next, the plant material was dried at 70°C to a constant weight, and then ground in a laboratory ball mill to homogenise the composition. The dried biomass was weighed with an accuracy of 0.01 g, and then subjected to the further examinations.

### Analytical methods

The physical and chemical parameters of the mixtures were determined using the following procedure: granulometric composition with sieve analysis method (PN-EN ISO 14688–2:2006) [31], specific gravity (ρ_s_), Le Chatelier flask method, at a temperature of 22˚C, bulk density (ρ_o_), Kopecky’s cylinder method with standard volume of 100 mL. Total porosity (n) was calculated on the basis of specific and bulk densities, according to the following equation: $n=\frac{\rho_{s}-\rho_{o}}{\rho_{s}}100\%$. The content of organic matter was determined by means of gravimetric methods, considering the loss of weight during calcination at the temperature of 550°C. The pH value was determined by means of the potentiometric method, in distilled water and in 1 M solution of potassium chloride (PN-ISO 10390: 1997 P) [32] and EC was measured for 1:5 soil/ water (weight/volume) according PN-EN 27888:1999 [33]. The tests of hydrolytic acidity (HA) and total exchangeable bases (TEB) were conducted by means of the Kappen method. The value of these parameters were used for calculation of cation exchange capacity (CEC) and base saturation (V), according to the formulas: CEC = HA + TEB, and V = 100% · TEB/CEC. Total organic carbon (TOC) was analyzed by means of a TOC-5050A Schimadzu analyzer and total nitrogen (TKN) content with the Kjeldahl method using 1002 Kjeltech distillation unit. Additionally, the samples were tested for the content of available phosphorus and potassium using the Egner-Riehm (DL) method.

### Statistical methods

The data obtained in the experiment were analyzed through parametrical test ANOVA and *post-hoc* Tukey’s test (letter indicators at the average content values indicate statistically homogeneous groups, called Tukey homogeneous groups; the presence of the same indicator designates the lack of a statistically significant difference between them), hierarchical cluster analysis (HCA), and principal component analysis (PCA) to evaluate the significant differences between the variables and correlation structure among the parameters measured. The analyzed variables were expressed in different units; therefore, the main components in the PCA analysis were calculated using the correlation matrix. All statistical tests were conducted by means of Statistica 2013 software package.

## Results

### Physical and physicochemical properties of soil-waste mixtures

It was observed that the drill cuttings addition to the soil increased the share of sand and decreased the share of silt fraction, while the percentage of clay has not changed significantly. Additionally, the drilling wastes addition did not significantly change the bulk density and specific gravity or the porosity of the obtained mixtures (Table 3). The higher changes in physical parameters were observed in the case of a mixture containing 15% of drilling wastes. The value of specific gravity increased from 2.57 g·cm^-3^ to 2.73 g·cm^-3^, the bulk density from 1.14 g·cm^-3^ to 1.16 g·cm^-3^, and the porosity from 55.45% to 57.69%, compared to non-modified soil.

**Table 3:** Physical properties of the soil and the mixtures (average value ± standard deviation).

Soil substrate	Granulometric composition			Specific gravity	Bulk density	Porosity [%]
	[%]					
	Sand fraction	Silt fraction	Clay fraction	[g·cm^-3^]	[g·cm^-3^]	
**Z–0**	23	71	6	2.57^a^ ± 0.08	1.14^a^ ± 0.01	55.45^a^
**Z–2.5**	27	68	5	2.56^a^ ± 0.01	1.13^a^ ± 0.05	55.80^a^
**Z–5**	30	65	5	2.61^a^ ± 0.03	1.14^a^ ± 0.01	56.30^a^
**Z–10**	32	63	5	2.59^a^ ± 0.02	1.20^a^ ± 0.01	53.73^a^
**Z–15**	52	42	6	2.73^a^ ± 0.01	1.16^a^ ± 0.01	57.69^a^

The drilling wastes addition increased the carbonate content, pH value and electrical conductivity in a statistically significant way as well as slightly raised the content of organic compounds and C:N ratio in the considered substrates (Table 4). The carbonate content in the soil-waste mixtures increased along with the dose of drilling wastes. The *post hoc* statistical analysis conducted by means of Tukey test indicated that 10% and 15% drill cuttings addition significantly increases the organic substance content in the mixture.

**Table 4:** Selected chemical properties of the soils and the mixtures (average value ±standard deviation).

Soil substrate	Carbonate	Organic matter	Total nitrogen	Total organic carbon	C/N	pH	EC
	[%]						[mS cm^-1^]
**Z–0**	0.36^c^± 0.02	1.9^b^± 0.1	0.06^a^± 0.01	1.09^a^± 0.01	18.2	4.16^d^± 0.02	0.33^e^± 0.04
**Z–2,5**	0.9^b^± 0.05	2.0^ab^± 0.1	0.06^a^± 0.01	1.40^a^± 0.01	23.0	6.66^c^± 0.02	3.22^d^± 0.03
**Z–5**	1.39^b^± 0.28	2.2^ab^± 0.1	0.06^a^± 0.01	1.30^a^± 0.01	21.6	6.83^c^± 0.10	5.03^c^± 0.04
**Z–10**	3.44^a^± 1.28	2.3^ab^± 0.1	0.06^a^± 0.01	1.50^a^± 0.01	25.0	6.98^b^± 0.10	6.99^b^± 0.07
**Z–15**	3.67^a^± 0.9	2.6^a^± 0.1	0.06^a^± 0.01	1.50^a^± 0.01	25.0	7.09^a^± 0.05	9.93^a^± 0.02

The drill cuttings addition to acidic soil (pH = 4.16) raised the pH of mixtures to the range of 6.5–7.2. The specific electrical conductivity, which is considered a measure of soil salinity, increased along with the dose of drilling wastes. In the mixtures with the lowest, 2.5% drill cuttings addition, the EC value increased tenfold in comparison to the control soil; in the mixtures with 15% drilling wastes addition, EC reached the highest value of almost 10 mS·cm^-1^ (Table 4).

### Sorption properties of soil-waste mixtures

**Fig 1:**
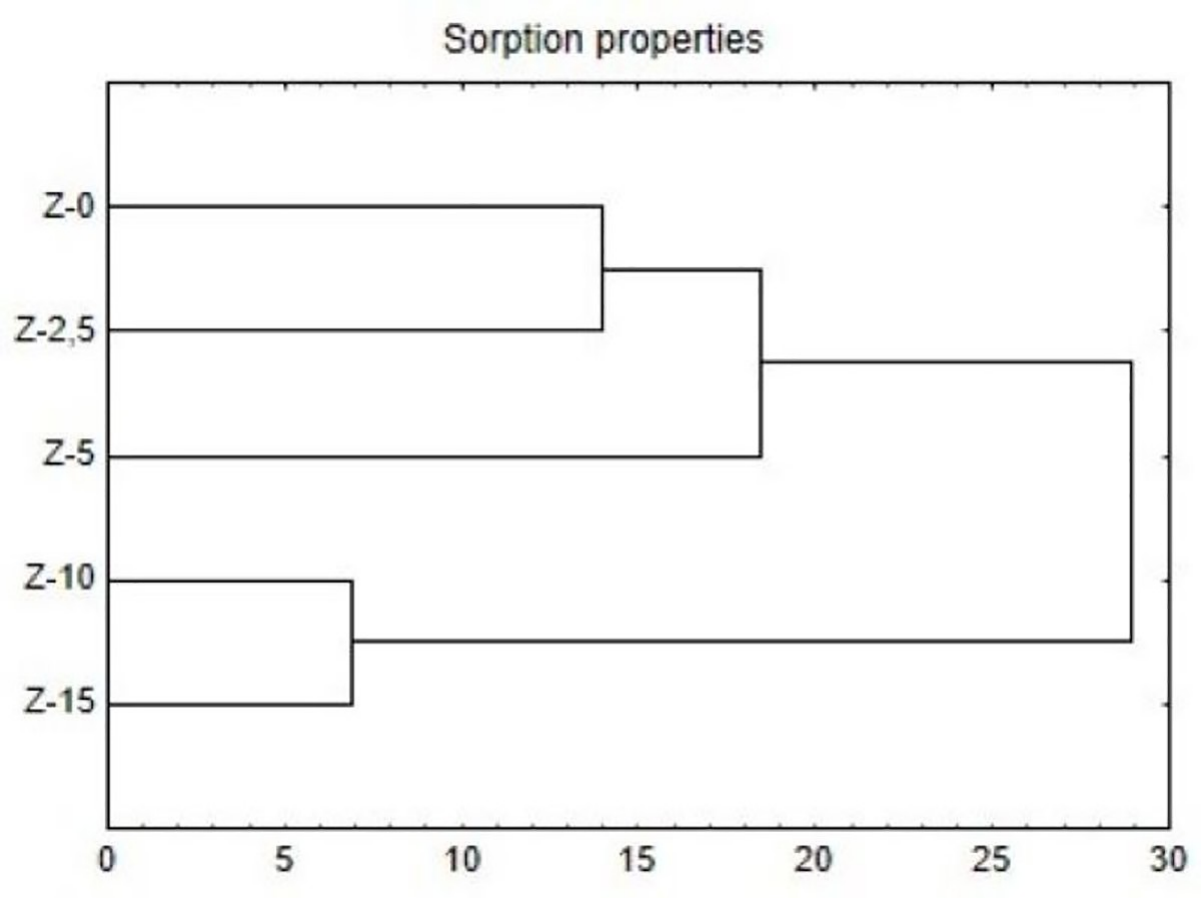
Dendrogram of the HCA results for sorption properties (CEC, TEB, and V) of the examined soil substrates.

The conducted studies indicate that highly alkaline drill cuttings had a statistically significant influence on the drop of hydrolytic acidity of soil-waste mixtures, which statistically significantly increased the base saturation value in comparison to the control sample. In the mixtures containing 2.5% and 5% of drilling wastes, the values of hydrolytic acidity were 15 time lower than in the control sample (Fig 1). The base saturation (V) of the exchangeable cation capacity in the mixture exceeded 97%, and these values were 3.5-fold higher than the ones measured in the control sample. The values of V in the mixtures did not differ statistically significantly from one another (Table 5). Along with an increase in the dose of the drill cuttings, a statistically significant increase in the content of TEB and CEC of mixtures was noted. The highest values of TEB (>46 cmol(+)/kg) and CEC (>7 cmol(+)/kg) were obtained in the substrates with the highest drill cuttings addition, i.e. the Z-10 and Z-15 mixtures, and these substrates were significantly different from other mixtures in this respect which was shown by the HCA analysis (Fig 1). In these mixtures, Ca^2+^ and Na^+^ were the dominant ions (Table 5).

**Table 5:** Effect of drill cuttings on exchangeable cations content and the degree of base saturation in s soil and mixtures (average value ± standard deviation).

Mixtures	Exchangeable cations				Base saturation
	Ca^2+^	Mg^2+^	Na^+^	K^+^	V
	[cmol (+)/kg]				[%]
**Z–0**	1.17 ± 0.03	0.74 ± 0.05	0.001	0.21 ± 0.05	26.43^a^
**Z–2.5**	11.44 ± 5.32	1.12 ± 0.14	0.26 ± 0.01	0.44 ± 0.03	97.07^b^
**Z–5**	23.64 ± 4.68	1.14 ± 0.15	0.41 ± 0.03	0.69 ± 0.06	97.88^b^
**Z–10**	43.73 ± 7.25	0.78 ± 0.02	0.84 ± 0.04	1.24 ± 0.14	97.48^b^
**Z–15**	47.48 ±7.65	0.92 ± 0.09	1.14 ± 0.05	1.62 ± 0.17	97.71^b^

One of the most important soil analyses is the assessment of available nutrients, i.e. phosphorus and potassium. The drilling wastes addition increased the content of available phosphorus forms in the examined mixtures. The drill cuttings addition enhanced the available phosphorus content but only to a limited degree up to the maximum value of 6.11 mg P_2_O_5_ 100 g^-1^ (Table 6).

**Table 6:** Available potassium and phosphorus in soil and the mixtures (average value ± standard deviation).

Mixtures	Content of potassium	Content of phosphorus
	[mg K_2_O 100 g^-1^ soil]	[mg P_2_O_5 _100 g^-1^ soil]
Z–0	2.18^a^ ±0.24	5.79^a^ ± 0.98
Z–2.5	5.11^b^ ± 0.94	6.11^b^ ± 0.17
Z–5	9.21^c^ ± 0.81	6.02^b^ ± 0.61
Z–10	16.85^d^ ± 0.51	6.09^b^ ± 0.97
Z–15	22.6^e^ ± 0.31	5.63^a^ ± 0.23

### Influence of drill cutting addition to the soil on the red cover growth

The differences in plant growth are a visible indicator reflecting the changes in soil quality. The drilling wastes addition to the soil had a positive influence on this indicator, regardless of the dose of wastes (Table 7).

**Table 7:** Mean values (and the standard deviation) of red clover dry biomass obtained in the pot experiment conducted on soil and soil-waste mixtures.

Mixtures	Biomass roots	Biomass shoot	Total biomass
	[g]		
**Z–0**	0.20^a^ ± 0.08	0.16^a^ ± 0.01	0.36^a^
**Z–2.5**	0.23^a^ ± 0.01	0.39^b^ ± 0.02	0.62^a^
**Z–5**	0.32^a^ ± 0.09	0.55^b^ ± 0.07	0.87^b^
**Z–10**	0.25^a^ ± 0.03	0.41^b^ ± 0.05	0.66^ab^
**Z–15**	0.28^a^ ± 0.09	0.27^c^ ± 0.04	0.55^a^

The highest clover biomass was obtained in the case of the Z–5 mixture, which contains 5% of drill cuttings (v/v) (Table 7). In comparison with the biomass of the plants which grew on the control soil, this value was over twice higher. In the remaining mixtures, the mass of plants was approximately 1.5-fold higher than the one obtained on the soil without the drill cuttings addition. The Tukey test did not show any statistically significant differences between the root mass of the clover cultivated on particular mixtures. However, there was a certain differentiation in the case of the clover shoot mass. The test also showed that the addition of drilling wastes significantly increased the clover shoot mass cultivated on all considered mixtures.

### Correlations between soil properties and plant growth

The PCA analysis was carried out in order to find the causes of the observed changes in the growth of clover biomass. On the basis of this analysis, the 20 variables were reduced to two principal orthogonal components (PC1 and PC2). PC1 is more strongly correlated with the variables–ten loadings have high values from 0.84 to 0.98 while in the case of PC2 only four loadings have a values from 0.76 to 0.87 (Table 8).

**Table 8:** Correlations between the principal components and the analyzed variables (p < 0.002).

No.	Variable	Principial component	
		PCA1	PCA2
1	Specific gravity	0.69	-0.46
2	Bulk density	0.54	-0.28
3	Porosity	0.21	-0.15
4	Carbonate	**0.91**	-0.34
5	Organic matter	**0.86**	-0.40
6	Total nitrogen	0.04	-0.04
7	Total organic carbon	**0.87**	0.21
8	C/N	0.69	0.17
9	pH	**0.90**	0.38
10	EC	**0.98**	0.02
11	Ca^2+^	**0.96**	-0.21
12	Mg^2+^	0.17	**0.80**
13	Na^+^	**0.94**	-0.31
14	K^+^	**0.93**	-0.34
15	Base saturation (V)	**0.84**	0.49
16	Biomass of roots	0.65	0.41
17	Biomass of shoots	0.42	**0.87**
18	Total biomass	0.51	**0.80**
19	Available K (K_2_O)	**0.93**	-0.35
20	Available P (P_2_O_5_)	0.06	**0.76**
Explained variance (%)		53.13	21.36
Cumulative (%)		53.13	74.49

The highest contributions of variables are highlighted in bold.

**Fig 2:**
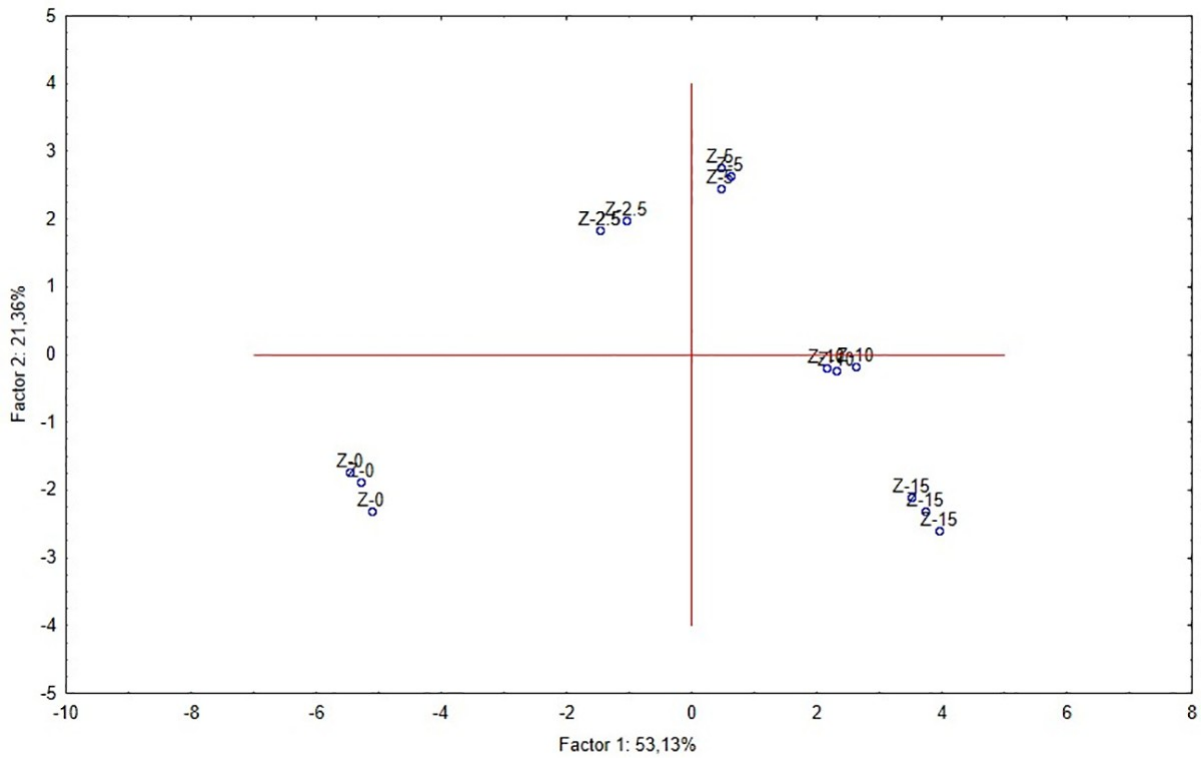
Projection of the variables of the factor plane. The correlation matrix is singular, so the generalized reciprocal method is used.

A factor loadings plot (Fig 2) illustrates the strength with which the variables influence the principal components and the variables are correlated between each other. It was stated that the chemical properties such as: EC, pH, content of carbonate, alkaline cations Ca^2+^, Na^+^, K^+^ (and K available form), Mg^2+^, organic matter, TOC, available P form and V, as also as total biomass and biomass of shoots explain *ca*. 75% of the variation. It can be observed that total biomass and shoots biomass are the most strongly correlated with the contents of Mg^2+^, available P, V and pH. Therefore, it can be assumed that low value of these parameters were factors limiting plant growth in the control soil.

**Fig 3:**
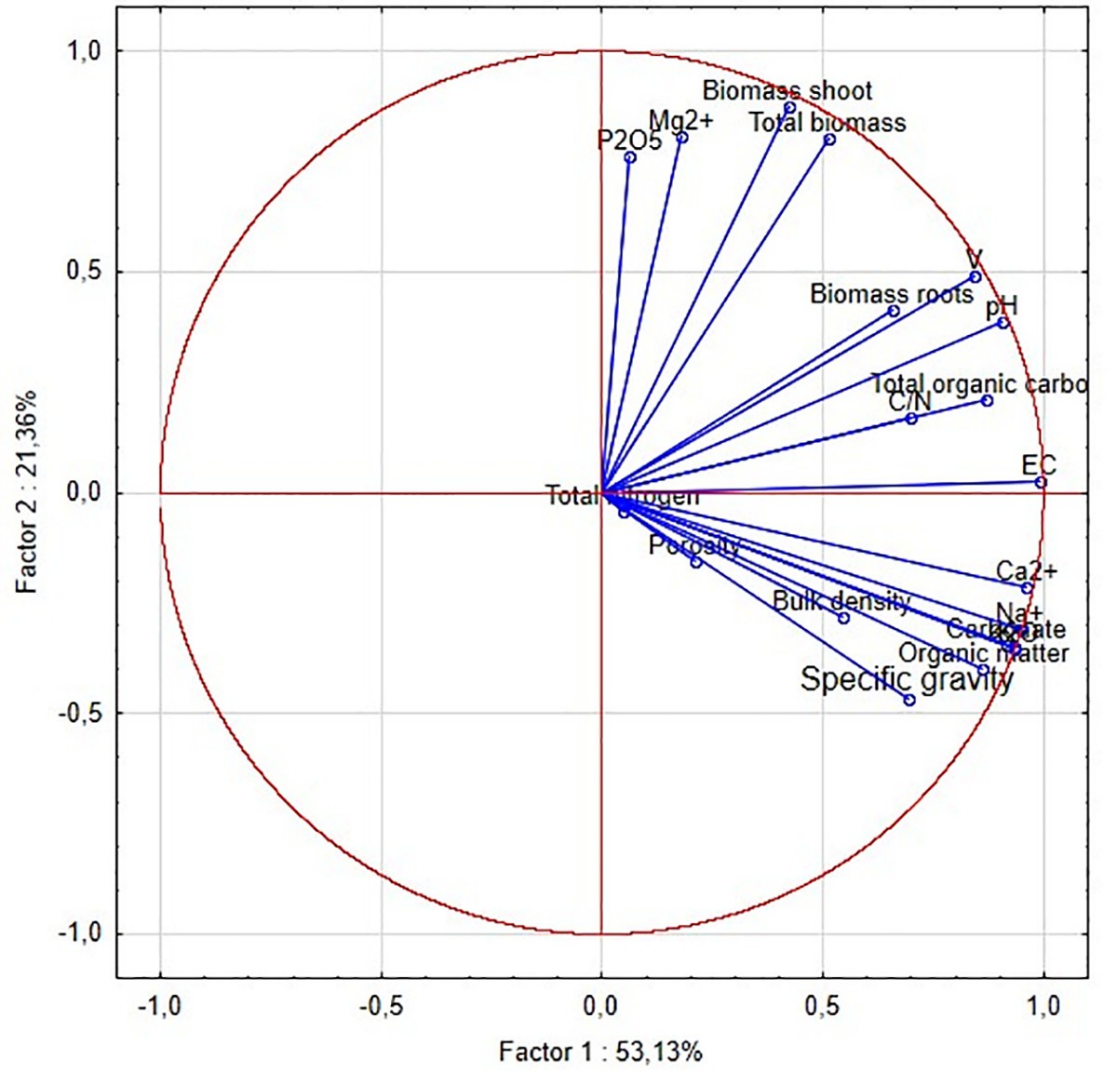
Projection of the cases on the factor plane. The correlation matrix is singular, so the generalized reciprocal method is used.

The control soil was the most different from all the examined materials while the Z-10 and Z-15 mixtures showed the lowest differences in terms of the variables related with PCA1 (Fig 3).

## Discussion

The research on the effect of the addition of drill cuttings on the properties of acid soils was undertaken in order to assess the legitimacy of using these wastes to improve the properties of soils on degraded areas, e.g. in the vicinity of sulfur mines or on the heaps of mining wastes. The high pH value and the base saturation are practical premises for such application. However, the basic principle of using wastes in recovery processes, which consist in incorporating them into the surface layer of the soil is founded on the statement that the wastes cannot contribute to the deterioration of the properties of the reclaimed soil. Taking into account the origin of drilling wastes, such a risk exists, which is the reason why the European regulations do not allow these wastes to be recovered it this way. However, the American and Canadian experiences show that the rational and recommended discharge of drilling wastes into the environment does not pose a threat to it.

In order to assess the risk related to the application of these wastes into the environment, the authors conducted the research on a material which is a mixture of drilling wastes of various origins and composition, collected from the drilling wastes final management facility, where the spend drilling muds with the cuttings generated during the oil and natural gas exploration in southern Poland are deposited. The examined wastes did not have the composition typical for the specific type of mud or drilled rock, which makes the evaluations and comparisons difficult.

Acidic soil was used in the experiment to simulate the properties of the material degraded with human activities. The content of macronutrients available for plants allowed classifying it to unfertile class [34]. Drilling wastes were added to the soil with gradually growing dose from 2.5 to 15% of the mixture weight (which correspond to 100–750 m^3^·ha^-1^ assuming the bulk densities of wastes and soil 1.1 and 1.4 t·m^-3^, respectively, and depth of soil layer of 0.3 m). All the doses were below the maximum rate of wastes approved for using in the landspreading method (050 Direcitve [35]) that is 1000 m^3^ ha^-1^ (which corresponds to *ca*. 20% of weight, assuming the same values as given above).

The conducted experiments showed that the drill cuttings addition altered the physical, chemical and physicochemical properties of the considered acid soil and influenced the red clover growth. An addition of drill cuttings increased the content of sand fraction in the waste-soil mixtures. However, only in the case of the substrate with the highest (15%) dose of drill cuttings, the sand fraction increased sufficiently to change the textural classification of soil from silt loam to sandy loam (in accordance with the USDA soil taxonomy [30]). However, even this change had no significant influence on the bulk density, specific gravity and porosity of the considered mixtures, although these parameters generally increased with the dose of the wastes. For example the highest increase in porosity, from 55.5% (in control soil) to 57.7% was observed in the case of mixture with 15% of wastes. This growth was less noticeable compared to the results of the study of Zvomuya et al. (2009) [21], in which total porosity of soil increased from 51% to 60% after adding of 80 m^3^·ha^-1^ (*ca*. 2% of weight) of spent water-based drilling wastes. However, the latter study was conducted on sandy loam which has more macropores than silty loam; thus, an increase in the content of fine fractions can strongly influence the total porosity of soil.

The addition of drilling wastes caused more visible changes in the chemical parameters of the soils. The content of organic compounds and organic carbon in the substrates increased along with the amount of drill cuttings in the mixtures that was confirmed by the results of the PCA analysis. Similar dependencies between the content of organic compounds and the increasing amount of drilling wastes in soils were noted by Kisic et al. (2009) and Bauder et al. (2005) [16, 17]. The highest concentration of organic compounds (2.6%) and organic carbon (1.5%) in our experiment were observed in the substrate that contained the highest (15%) dose of drill cuttings. These values were approximately 1.3-fold higher than in the control sample; however, they were still lower than those recommended for agricultural soils, that are 2–2.6% for organic matter and 1.3–1.5% for TOC [36]. However, it should be noticed that the organic compounds supplied with drill cuttings are qualitatively different from the soil organic matter, because it mainly originated from drilling fluids. Thus, their role is not always positive. Some organic compounds used in drilling fluids may unfavorably influence the soil processes and TOC increase in the soil enriched with drilling wastes should be controlled. Ball et al. (1996) indicated that the application of drilling wastes containing such organic compounds as starch and xanthan gum stimulates the denitrification processes, which may deplete the nitrogen in soils [37]. The authors did not know the composition of the muds contained in the examined wastes, thus the risk of nitrogen release could not be anticipated, but even without this phenomenon the examined soil-waste mixtures were very poor in this element. Both the soil and drilling wastes used in the study contained trace amounts of nitrogen (0.06 and 0.04%, respectively). Thus, the addition of drilling cuttings did not significantly change the content of this element in the soils. Yao et al., (2014) even observed a decrease in the available nitrogen content in the loam soils mixed with the spent potassium drilling muds with the dose 120 m^3^·ha^-1^ [14]. The drilling wastes are usually poor in nitrogen [37] and they cannot be considered as a source of this element; thus, an additional source is needed in the unfertile marginal soils. Additionally, too high C:N ratios in the examined mixtures, which ranged from 21.6 to 25, indicate that nitrogen could be sufficiently available for plants. When the C:N ratio of soil organic matter is below 15, rapid mineralization and release of N occurs, and it is available for plant uptake [38]. A well-known source of the nitrogen for soil are legumes (*Fabaceae*). These plants live in symbiosis with the *Rhizobium* bacteria that are able to fix the atmospheric nitrogen (N_2_) and convert it into the plant available forms. Therefore, the red clover (*Trifolium pretense* L.) was used as a test plant. The high resistance to soil salinity is the second important advantage of this species, because the drill cuttings addition caused the increase in EC of the soil-waste mixture. While comparing the EC values measured in the investigated mixtures with the Jackson’s salinity scale (Jackson, 1967) [39] and accounting for the relevant dilution of the samples, it was stated that the drill cuttings addition resulted in a change of the soil category from “non-saline” in the control variant (EC<1.6 mS/cm), through “mildly saline” (EC 1.6–3.2 mS/cm) for the mixture containing 2.5% (w/w) of drill cuttings and “moderately saline” (3.2–6.4 mS/cm) in the variant containing 5% (w/w) of wastes, to “highly saline” (>6.4 mS/cm), in the case of the substrates containing 10% and 15% (w/w) of drill cuttings. Taking into account the plant requirements in terms of the EC value of soil, it can be stated that the variant containing 2.5% of drill cuttings should not exert a negative influence on the growth of plants which do not exhibit the susceptibility to salinity. The mixtures containing 5% and 10% of wastes may limit the plant growth to a varying degree and the substrate with the highest drill cuttings dose (Z–15) may be successfully inhabited only by halophytes. The red clover, which is considered as saline tolerant plants [40], proved to be resistant even to highly saline level, in which the total biomass was over 1.5-fold higher than in the control, non-saline soil. The PCA analysis did not show the significant inhibiting effects of EC value, while it indicated the important influence of pH on the red clover growth. The value of pH increased significantly with the drilling wastes dose from pH 4.2 in the control soil to pH 6.66–7.09 in the soil-wastes mixtures. Thus, the addition of drill cuttings to the soil resulted in changing the substrate category from “acidic” to “slightly acidic” or “neutral” according to scale given by Jackson (1967) [39], or from extremely acid to neutral, according to the US soil standards [30]. The values obtained in the mixtures are considered optimal for the plant growth because they favour the availability of microelements [41], which was a reason of the increase in plant biomass.

The increase in pH can be attributed to the increase in carbonates content with drill cuttings addition to the values which many times exceeded the average carbonate content noted in the Polish soils, amounting to 0.46% [42]. In the mixture with 2.5% of drill cuttings addition, the concentration of carbonates was more than twice higher than in the control soil, while in the mixtures containing 10% and 15% of drill cuttings, the content of carbonates increased ten times. The elevated carbonate content resulting from drill cuttings addition was observed also by Jamrozik et al. (2009) [15] in the soil enriched with drilling wastes in the dose 5–40% of weight. The elevated carbonate content in drilling wastes is connected with the composition of drilling muds, in which calcium carbonate is used to increase their density [16], but it may also result from the presence of carbonates in the drilled rock.

The PCA analysis showed that the contents of alkaline elements: Ca, Mg, K, and Na were important variables contributing the differentiation of the mixtures with different dose of drilling wastes. Addition of 2.5% of drilling wastes increased the content of calcium by 20-fold, magnesium and potassium by 2-fold, as well as sodium by 200-fold. Despite the important quantitative changes, the share of particular exchangeable cations in the mixtures containing 2.5% and 5% of drill cuttings was typical for soils [43], and the amount of cations decreased in the following order: Ca^2+^>Mg^2+^>K^+^>Na^+^. Only an increase in the drill cuttings dose to 10% and 15% resulted in the disruption of this order, changing it to the following sequence: Ca^2+^>K^+^>Na^+^>Mg^2^. Although these elements can be considered as water-soluble and plant-available forms (their content was determined by the use the ammonia chloride for desorbing the cations from the solid phase), only the concentration of Mg^2+^ turned out to be strongly positively correlated with red clover biomass, which was confirmed by results of PCA. The concentration of Mg^2+^ in the examined mixtures were similar to these obtained by Gonet et al. (2006) [14]. For example, in their study, 1.18 cmol(+)/kg of magnesium was found in the soil that contained 10% of drilling wastes. Moreover, the concentration of other elements: calcium 44.1 cmol(+)/kg, potassium 0.27 cmol(+)/kg potassium and sodium 2.15 cmol(+)/kg were in a similar range compared to our results.

The drilling wastes additions influenced the soil in terms of the available forms of phosphorus and potassium. The soil used in the experiment (which it classified to heavy soils in terms of its grain size distribution) belongs to class V, in terms of the available potassium content, i.e. soils with very low available potassium content (less than 10 mg K_2_O 100 g^-1^) and to class IV in terms of the available phosphorus content, i.e. soils with low content of available phosphorus forms [42]. However, when considering the content of potassium, the higher soil class was achieved only in the mixtures with the dose of 10% and 15% of drilling wastes. According to this parameter, the Z-10 mixture was classified to class IV, i.e. substrates with low content of available forms of the considered element (ranging from 10.1 to 15.0 mg K_2_O 100 g^-1^ of soil), and Z-15 mixture was classified to class III, i.e. soils with moderate content of available potassium forms (within the range of 15.1–25 mg K_2_O 100 g^-1^ of soil) [42]. The concentration of available phosphorus was only slightly increased due to the drill cuttings addition (up to the maximum of 6.11 mgP_2_O_5_ 100 g^-1^). The low value of available phosphorus forms may stem from the high carbonate content (0.9%–3.67%), which increases the chemisorptions of phosphorus [12]. Similar results were observed by Yao et al. (2014 [18]). In their study, the addition of spent potassium drilling muds to loam soil did not result in a marked increase of the available phosphorus forms either. In turn, Zvomuya et al. (2011) observed an elevated concentration of available phosphorus forms in the soils supplemented with drilling wastes in the form of sludge, but only after 3 years of their application [22]. This time dependent effect may be explained by slow decomposition rate of phosphorus compounds. Additionally, the changes in soil chemical status over the time might cause desorption of phosphate ions that were previously strongly adsorbed by soil components. What is important, a slight increase in the available phosphorus concentration in our study did not change the classification of the soil, but the PCA results indicate that it was significant factor influencing the plant growth.

The PCA and cluster analysis showed that the sorption parameters contributed to significant differences between the examined soil mixtures. The drilling wastes addition increased the CEC value from 2-fold (in the case of Z-2.5) to 6-fold (in the case of Z-15). However, the soil with 2.5% drill cuttings addition was the most similar to non-modified soil, and soil which contained 10% was the most similar to that with 15% of drill cuttings. While comparing the CEC values determined in the mixtures containing drill cuttings with the values of this parameter given in the Lityński (1976) [44] scale used for the soil quality assessment, a very strong cation exchange capacity (CEC>9.0 cmol(+)/kg) was found. In turn, the control soil was characterized by a strong cation exchange capacity (7.6≤T<9.0). Enhancement of the CEC in the soil caused by the drill cuttings addition was also confirmed in the other studies conducted on sandy soils [14].

Variations in the biomass growth evidently indicate the differences in soil quality. A significant increase (from 1.5 to 2.5-fold) in total biomass and shoots biomass compared to the control substrate were observed in all the soil-waste mixtures, which proves the improvement in the conditions of red clover (*Trifolium pretense* L.) growth and indicates the suitability of the examined drilling wastes in acidic soil remediation. However, the highest amount of red clover biomass was obtained in the mixture containing 5% of drilling wastes. An increase in the biomass growth due to drilling wastes addition was also observed by other researchers. They found significantly lower doses that stimulated the plant growth. Yao et al. (2014) found that the biomass of barley (*Hordeum vulgere* L.) and grass (*Agropyron trachycaulum*) increased *ca*. 1.8-fold when the spent potassium drilling mud was added to the sandy soil with dose 60 m^3^ ha^-1^ (which corresponds to *ca*. 1.5% of weight) [18]. Magalhães et al. (2014) found the beneficial influence of doses of 0.03% - 0.3% of weight of the oil-based drill cuttings addition to the soil on rice (*Oryza sativa*) growth, but increasing the dose to 0.6% inhibited the plant growth [29]. The authors attributed this phenomenon to an increase in EC, exchangeable sodium concentration, and solubilization of barite due to low redox potential in rice field, but they did not analyze the concentration of PAHs and other organics which probably were contained in the residues of oil-based muds and may also exert toxic effect on plants. Bauder et al. (2005) and Zvomuya et al. (2011) did not find the positive effect of drilling wastes addition on the plant growth but they determined the safe dose that did not deteriorate the soil conditions and negatively influence the growth. According to Bauder et al. (2005) no negative influence on the growth of winter wheat (*Triticum aestivum* L.) [16] was observed for spent bentonic drilling muds spread at the dose below 94 Mg·ha^-1^ (which corresponds to 2.2% of weight when the cuttings were used). According Zvomuya et al. (2011) the safe dose for spent water-based drilling wastes used in the landspraying method was lower than 20 m^3^·ha^-1^ (which corresponds to 0.5% of weight when the cuttings were used) [22]. The negative impact of drilling wastes, applied even in low doses was shown by Gonet et al. (2006). In their experiment, already a 5% drilling wastes addition to brown soil resulted in the reduction of the red fescue yield (*Festuca rubra*) by 30%, while 40% wastes addition resulted in over 50% decrease in these plant yield [14]. These divergent results indicate that selecting a dose of drilling wastes which could improve the soil properties and increase plant yield, or at least do not contribute to deterioration of these parameters, requires a case study. The results depend not only on the type of wastes, the method of its application, but also on the properties of the soil into which it is applied. The soil properties seems to be very important. Addition of wastes, even in low amount, to the fertile soils e.g. brown soil, may worsen their parameters and reduce the plant growth, as indicated in the studies by Gonet et al. 2006 [14], while in the case of poor quality soils with a low content of macro- and microelements, the wastes application may have a positive effect, as evidenced by the results of our research.

## Conclusions

The results of the study showed that drilling waste, that are not classified to hazardous one, when applied in relevant doses, can be managed by using for reclamation of degraded lands. The addition of the mixed drill cuttings in doses 5 to 15% of weight to the poor with nutrients, acidic loamy soil improved the properties of the soil, especially in terms of pH and the content of alkaline cations, as well as increased the yield of red clover biomass even 2.5 times. However, the stimulating effect on plant growth was gradually reduced when the wastes dose exceeded 5% of the mixture weight.

From a practical point of view, due to the high pH and buffer capacity, the wastes could be used for the reclamation of land degraded by acidification, e.g. located in the vicinity of opencast sulfur mines, on heaps of mining wastes containing acid-forming minerals, e.g. pyrite. However, the selection of plant species used in the reclaimed area should take into account their resistance to salinity, as the addition of waste materials significantly increases the EC value and the concentration of alkaline cations in the soil. The study has shown that red clover is a suitable plant species for such soils, not only because of its high tolerance to EC, but also due to its ability to fix nitrogen from the air. This feature is important because both in acidic soils and drill cuttings, the content of this element is low.

In order to minimize the risk of contamination of groundwater and increase the possibility of bioremediation (related to the accumulation of metals in the biomass), it is recommended to use drilling cuttings in a dose of 5%, which turned out to be the most beneficial for the red clover growth and did not contribute to strong soil salinity. This dose corresponds to the addition of *ca*. 200 m^3^ of drill cuttings per hectare. The wastes should be distributed on the ground non-covered with vegetation, mixed with a surface soil layer of at least 0.3 m, and then sown with the clover. The intensified increase in growth of the above-ground biomass allows using the plants for soil bioremediation. However, there is a risk of excessive accumulation of heavy metals or barium in plant shoots; therefore, their further management should be preceded by the relevant chemical analysis. Groundwater monitoring in the reclaimed area is also needed, as there is a risk of migration of dissolved substances, including heavy metals from the upper to the underlying soil layers.
